# Regulatory Machinery of Bacterial Bioflocculant Synthesis and Optimisation and Assessment of Bioflocculation Efficiency in Wastewater

**DOI:** 10.3390/ijms262110559

**Published:** 2025-10-30

**Authors:** Stanley Mokoboro, Tlou Nelson Selepe, Tsolanku Sidney Maliehe, Kgabo Moganedi

**Affiliations:** 1 Department of Biochemistry, Microbiology and Biotechnology, University of Limpopo, Private Bag X1106, Polokwane 0727, South Africa; 201826400@myturf.ul.ac.za; 2 Department of Water and Sanitation, University of Limpopo, Private Bag X1106, Polokwane 0727, South Africa; tlou.selepe@ul.ac.za; 3 Department of Biochemistry, Genetic and Microbiology, University of KwaZulu Natal, Private Bag X 54001, Durban 4000, South Africa; maliehet@ukzn.ac.za

**Keywords:** *Klebsiella michiganensis*, *Klebsiella pasteurii*, gene clusters, bioflocculation, optimisation, wastewater treatment

## Abstract

Bacteria are promising sources of bioflocculants, yet their regulatory machinery for bioflocculant synthesis remains underexplored. This study focused on evaluating the biosynthetic genes, optimisation and assessment of bioflocculation efficiency in wastewater. The isolated bioflocculant producers were identified by 16S rRNA and *rpo*B gene analysis. Polymerase chain reaction was used to assess the presence of polyketide synthase I (PKS-1), polyketide synthase II (PKS-II), non-ribosomal peptide synthetase (NRPS), epsH and epsJ. A one-factor-at-a-time technique was utilised for optimisation of culture conditions. The bioflocculants’ efficiencies were evaluated in wastewater using the Jar test method. Among 31 isolates, *Klebsiella michiganensis* and *Klebsiella pasteurii* were the most potent bioflocculant producers. They both revealed the presence of PKS-II. *K. pasteurii* possessed the epsH gene. The optimal conditions for maximum bioflocculant production (95% activity) by *K. michiganensis* were a temperature of 35 °C, pH of 5, galactose, tryptophan and 84 h of incubation. *K. pasteurii*’s maximum bioflocculant production of 83% was obtained at a temperature of 35 °C and pH of 7, with galactose, a mixture of urea, yeast extract, and ammonium sulphate (NH_4_)_2_SO_4_ and 96 h of fermentation. Their bioflocculants reduced the chemical oxygen demand and turbidity of wastewater by more than 70%. The bacteria had promising bioflocculant production with potential applicability in wastewater treatment.

## 1. Introduction

Bioflocculants are natural flocculants secreted by plant (e.g., tannins and plant extracts), animal (chitosan) and microbial cells (carbohydrates, proteins, lipids and glycoproteins) as a defence mechanism against biotic and abiotic stress [1]. They are non-toxic, biodegradable and innocuous to humans and produce zero secondary pollution [2]. Microbial flocculants, biopolymers secreted by microorganisms such as fungi and bacteria during their growth, have gained significant attention [3]. This is because they are easily reproducible and are consistent in quality as they are less affected by climate change, as opposed to plants [4]. Amongst the identified microbial flocculant producers, bacterial strains are the most common producers, underscoring their high metabolic capabilities for producing bioflocculants [5,6].

The low yields of bacterial bioflocculants have been one of the major drawbacks for their industrial application [7]. To combat this challenge, screening of novel strains with the ability to produce high yields with profound activities remains the primary strategy for mitigating the challenge of low yields [8,9]. Although this strategy is effective to some extent, the results of neglecting to explore regulatory bioflocculant-production mechanisms, such as biosynthetic functional genes, enzymes and pathways, cannot be overstated [10]. It is clear that it is impossible to ascertain the key reasons for low bioflocculant yields by only exploring the effects of environmental conditions and medium composition. Hence, over the past decades, there has been a stagnation in the improvement of bioflocculant yields.

The biosynthesis of bioflocculants is mediated by the expression of flocculation-related biosynthetic gene clusters (BGCs) and genes [11]. BGCs include genes encoding tailoring enzymes such as oxidoreductases, methyltransferases, acyltransferases, and glycosyltransferases that modify the secondary metabolites’ skeletons to enhance their functionality [12,13,14]. They comprise genes encoding enzymes responsible for building the molecular backbone of different classes of secondary metabolites, for example, non-ribosomal peptide synthetase (NRPS) enzymes and polyketide synthases (PKSs) [15]. Therefore, exploration and manipulation of genes and enzymes expressed during bioflocculant production and identification of biosynthesis pathways can increase the conversion efficiency of microbial substrates, consequently enhancing bioflocculant production. Moreover, the identification of flocculation-related genes can aid in the construction of highly efficient engineered bacterial strains, consequently overcoming the uncertainties of low yields, promoting reproduction of the bioflocculants [16].

The genomic information of most bioflocculant-producing strains remains unknown, which subsequently limits the identification of key enzymes and metabolic pathways involved in bioflocculant synthesis [17,18]. To date, functional genes encoding for bioflocculant production have been identified in a few bacteria. Among the promising bacterial bioflocculant producers, *Bacillus licheniformis* is the most explored and best-understood bacterium. The whole genomes of 17 *B. licheniformis* strains have been comprehensively studied [13]. An eps operon consisting of 16 genes involved in polysaccharide biosynthesis has also been identified in *Bacillus subtilis* [19]. The presence of exopolysaccharide biosynthesis genes was recognised in *Lactobacillus helveticus* [20] and *Xanthomonas campestris* [21]. Comstock et al. [22] have discovered the *wcf* gene cluster, which consists of *wca*fA-L, *wzy*, and *wzx*, which are responsible for extracellular polysaccharide biosynthesis. Thus, identification of BGCs and enzymes responsible for EPS (bioflocculant) production can provide crucial insights into the molecular basis of bioflocculant synthesis and has the potential to improve bioflocculant yield through their manipulation.

This study aimed to isolate bioflocculant-producing bacteria and optimise their culture conditions for the enhancement of bioflocculant production. Moreover, we explored the presence of different flocculation-related genes and gene clusters to ascertain the mechanisms involved during bioflocculant synthesis.

## 2. Results

### 2.1. Isolation and Identification

A total of 31 bacterial isolates were obtained from recreational ponds and screened for their capability to produce bioflocculants. Out of 31 obtained isolates, only isolates MS7 and MS18 exhibited a flocculating activity of over 50%, with MS7 having 58 ± 0.04% and MS18, 53 ± 0.03%. Isolate MS7 shared 99.42% of its identity with *Klebsiella michiganensis* (accession number PX353943), while Isolate MS18 exhibited a 99.54% similar identity with *Klebsiella pasteurii* (accession number PP809651.1).

### 2.2. Cluster Genes Responsible for Bioflocculant Production

**Figure 1:**
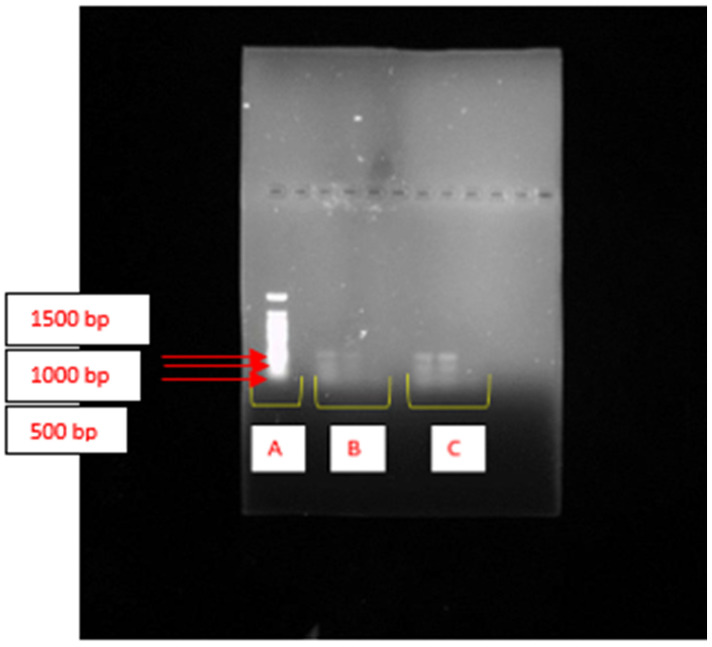
Agarose gel electrophoresis showing the amplification of the *KSα* gene cluster encoding PKS-II in *K. pasteurii* PP809651.1 and *K. michiganensis* PX353943. (**A**) represents the lane of the DNA ladder. (**B**) displays DNA extracted from *K. michiganensis* PX353943, while (**C**) shows DNA extracted from *K. pasteurii* PP809651.1 in triplicate.

PKS-1 and NRPS were not detected in both *K. pasteurii* PP809651.1 and *K. michiganensis* PX353943. However, the *KSα* gene cluster, which encodes for PKS-II, was found present in both *K. pasteurii* PP809651.1 and *K. michiganensis* PX353943, as shown by the presence of the illuminating bands on the gel in Figure 1.

### 2.3. Enzymes Coded by Genes Responsible for Bioflocculant Production

**Figure 2:**
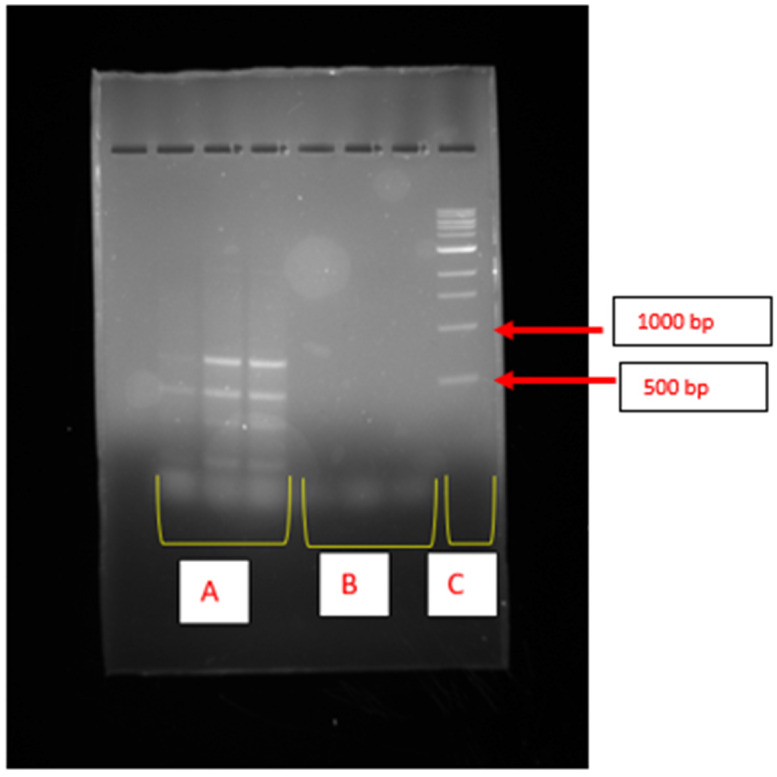
Agarose gel electrophoresis showing the amplification of the epsH gene encoding glycosyltransferase enzymes. (**A**) illustrates the DNA extracted from *K. pasteurii* PP809651.1 in triplicate. (**B**) includes the lanes corresponding to DNA extracted from *K. michiganensis* PX353943, whereas (**C**) represents the lane of the DNA ladder.

The epsH gene, which encodes a glycosyltransferase enzyme, was only detected in *K. pasteurii* PP809651.1, as illustrated by the illuminating bands on the corresponding gel (Figure 2A). The illuminating bands on the gel lanes of *K. michiganensis* PX353943 were the dimmest and indicated no amplification of the epsH gene (Figure 2B). Figure 2C indicates the biomarker, which was used as a reference.

### 2.4. Optimisation of Conditions

#### 2.4.1. Inoculum Volume

**Figure 3:**
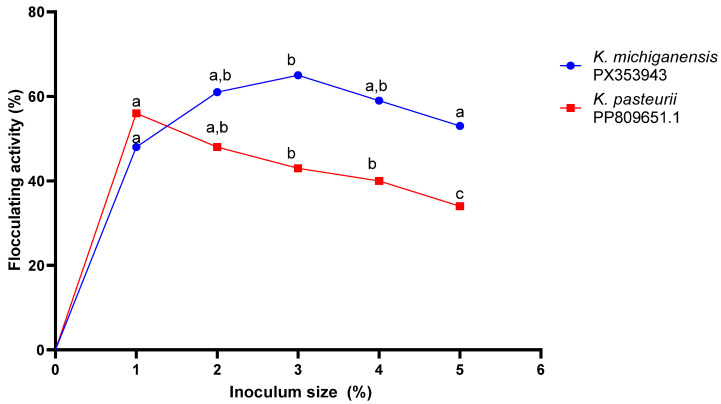
The effect of inoculum size on bioflocculant production. The letters (a, b, c) denote statistical significance at *p* < 0.05. Values sharing the same letter along the line are not significantly different (*p* > 0.05).

Figure 3 below shows the effect of inoculum size on bioflocculant production. For *c*, there was an insignificant increase (*p* > 0.05) in bioflocculant production as indicated by the flocculating activity from inoculum size 1% to 3%. The highest bioflocculant production (65 ± 0.03% FA) was obtained at an inoculum size of 3%. However, the inoculum size of 2% was selected for subsequent experiments. Likewise, there was a constant decrease in bioflocculant production with an increase in inoculum size from 1 to 5% for *K. pasteurii* PP809651.1. The highest bioflocculant production, as shown by a flocculating activity of 56 ± 0.003%, was observed when an inoculum size of 1% was used. Thus, a 1% inoculum size was used in the subsequent assays.

#### 2.4.2. Carbon Sources

Table 1 illustrates the effect of nutritional sources on bioflocculant production by *K. michiganensis* PX353943 and *K. pasteurii* PP809651.1. Amongst the carbon sources investigated, both bacterial strains preferred galactose as a carbon source, yielding the peak bioflocculant production with flocculating activities of 74 ± 0.025 and 79 ± 0.008%, respectively. *K. michiganensis* PX353943 poorly utilised sucrose and gave its lowest bioflocculant production with a flocculating activity of 56 ± 0.022%. *K. pasteurii* PP809651.1 assimilated lactose inadequately, resulting in its lowest bioflocculant production with a flocculating activity of 44 ± 0.007%. Therefore, in all the subsequent experiments, galactose was used as a carbon source for both strains.

#### 2.4.3. Nitrogen Sources

Different nitrogen sources were assessed on their effect on bioflocculant production as shown in Table 1. The strain *K. michiganensis* PX353943 showed a maximum bioflocculant production with a flocculating activity of 80 ± 0.043% when tryptophan was used as a source of energy. Contrarily, the bacterium had its lowest bioflocculant production with a flocculating activity of 13 ± 0.003% when urea was used as a source of energy. Furthermore, *K. michiganensis* PX353943 effectively utilised the mixture of nitrogen sources (urea, yeast extract, and (NH_4_)_2_SO_4_) and (NH_4_)_2_SO_4_ as a source of energy. Hence, there was an insignificant statistical difference (*p* > 0.05) between the mixture of nitrogen source and tryptophan. However, tryptophan was used in subsequent assays due to its abundance availability in the laboratory. The strain *K. pasteurii* PP809651.1 showed its highest bioflocculant production with a flocculating activity of 79 ± 0.01% when the mixture of nitrogen sources (urea, yeast extract, and (NH_4_)_2_SO_4_) was used. The lowest bioflocculant production, with a flocculating activity of 35 ± 0.025%, was obtained when yeast extract was used. Furthermore, the bioflocculant production obtained when the mixture of nitrogen sources was used was significantly high (*p* < 0.05). Therefore, the mixture of nitrogen sources was used in the subsequent experiments due to their abundant availability in the laboratory.

#### 2.4.4. Initial pH

**Figure 4:**
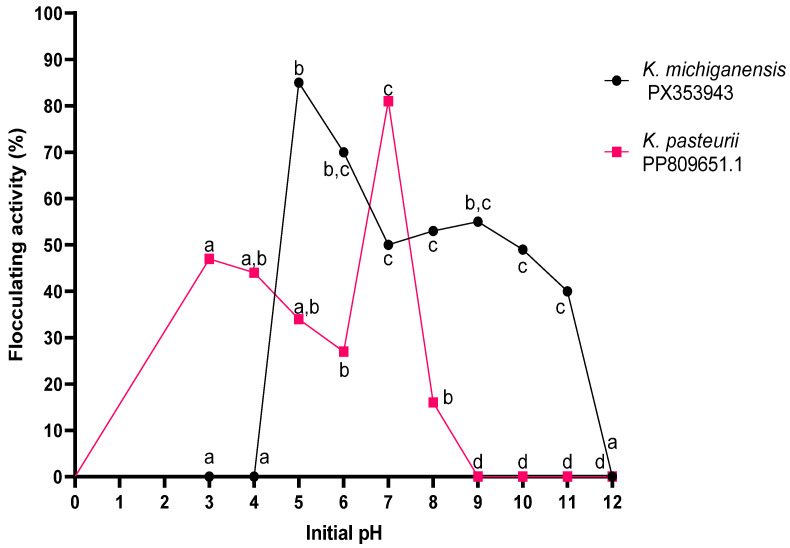
The effect of initial pH on bioflocculant production. The letters (a, b, c, d) denote statistical significance at *p* < 0.05. Values sharing the same letter along the line are not significantly different (*p* > 0.05).

The effect of initial pH on bioflocculant production for *K. michiganensis* PX353943 and *K. pasteurii* PP809651.1 was investigated and the results are shown in Figure 4. The bacterial strain *K. michiganensis* PX353943 showed optimal growth at pH range 5–6. Hence, there was an insignificant statistical difference (*p* > 0.05) between pH 5 and 6. The highest bioflocculant production, with a flocculating activity of 85 ± 0.024%, was attained at a pH of 5 whereas the lowest bioflocculant production, with a flocculating activity of 0%, was obtained at a strongly acidic pH range of (3–4) and at a strongly basic pH of 12. Thus, the production medium was adjusted to pH 5 for all the subsequent experiments. There was a fluctuation in bioflocculant production by *K. pasteurii* PP809651.1 within pH (3–8); ultimately, the bioflocculant production dropped and remained stagnant from pH 9 to 12. The peak bioflocculant production with a flocculating activity of 81 ± 0.016% was achieved at a neutral pH of 7. Hence, the production medium was adjusted to pH 7 for all the subsequent experiments.

#### 2.4.5. Temperature

**Figure 5:**
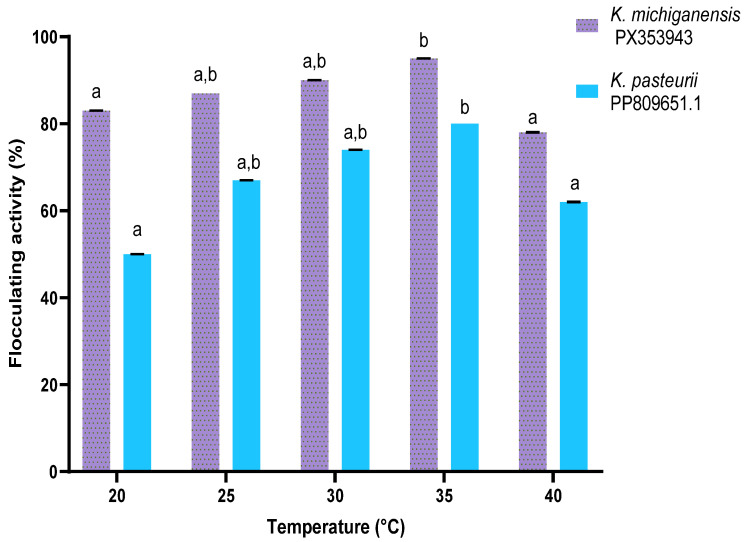
The effect of temperature on bioflocculant production. The letters (a, b) denote statistical significance at *p* < 0.05. Values sharing the same letter above the bars are not significantly different (*p* > 0.05).

Figure 5 depicts the effect of temperature on bioflocculant production by *K. michiganensis* PX353943 and *K. pasteurii* PP809651.1. The bioflocculant production of the bacterial strain *K. michiganensis* PX353943 increased insignificantly (*p* > 0.05) with the increase in temperature from 20 to 30 °C. The highest bioflocculant production with a flocculating activity of 95 ± 0.019% was attained at 35 °C. Subsequently, there was a significant (*p* < 0.05) decrease in bioflocculant production with the increase in temperature above 35 °C. Similarly, with the strain *K. pasteurii* PP809651.1, bioflocculant production increased insignificantly (*p* > 0.05) with the increase in temperature from 20 to 30 °C. The highest bioflocculant production, with a flocculating activity of 81 ± 0.019%, was attained at 35 °C. Thereafter, there was a significant (*p* < 0.05) decrease in bioflocculant production with the increase in temperature from 35 to 40 °C. Therefore, 35 °C was used as the incubation temperature for both strains in all subsequent experiments.

#### 2.4.6. Effect of Incubation Time on Bioflocculant Production

##### Effect of Time on Bioflocculant Production by *K. michiganensis* PX353943

**Figure 6:**
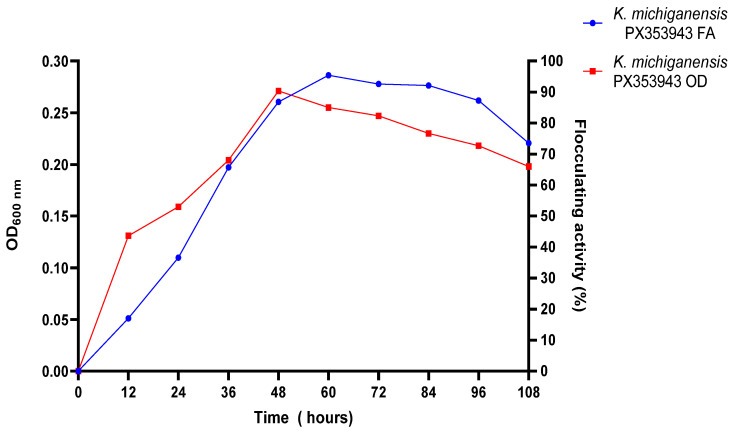
The effect of incubation time on bioflocculant production and growth of *K. michiganensis* PX353943. FA illustrates bioflocculant production, and OD indicates growth.

Figure 6 illustrates the effect of culture time on the bioflocculant synthesis of *K. michiganensis* PX353943 and its cell growth. A direct proportional relationship was observed between bioflocculant production and growth, indicated by the optical density (OD) of the bacterium. There was an increase in bioflocculant production by *K. michiganensis* PX353943 and its growth with an increase in incubation time from 12 to 48 h. The peak bioflocculant production, with a flocculating activity of 95%, and peak OD of 0.271 were obtained, respectively, after 60 h and 48 h of incubation time. After 60 h, the bioflocculant production and growth (OD) decreased steadily from 95% FA to 73% FA and 0.271 to 0.198, respectively.

##### Effect of Time on Bioflocculant Production by *K. pasteurii* PP809651.1

**Figure 7:**
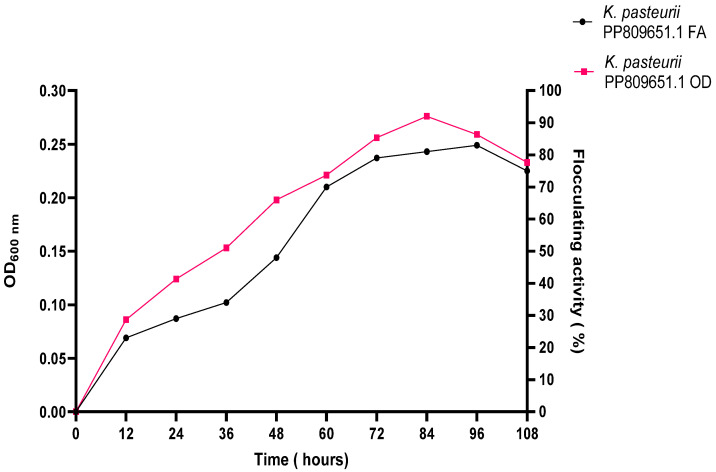
The effect of incubation time on bioflocculant production and growth of *K. pasteurii* PP809651.1. FA illustrates bioflocculant production, and OD indicates growth.

Figure 7 depicts the effect of culture time on the bioflocculant production and cell growth of *K. pasteurii* PP809651.1. Both bioflocculant production and OD, indicative of growth, increased progressively with incubation time between 12 and 84 h. The maximum bioflocculant production, with an FA of 83%, was achieved at 96 h, while the highest cell growth, reflected by an OD of 0.276, was observed at 84 h. Thereafter, a decline in OD and bioflocculant production was noted at 84 and 96 h, respectively.

### 2.5. Bioflocculant Yields

*K. michiganensis* PX353943 yielded 1.8 g/L, whilst *K. pasteurii* PP809651.1 produced 7.9 g/L of the bioflocculants. The bioflocculants were then designated BF-PX353943 for *K. michiganensis* and BFMS18 for *K. pasteurii* BF-PP809651.1.

### 2.6. Application of Purified Bioflocculants in Wastewater Treatment

The removal efficiencies (RE) of the flocculants are displayed in Table 2. Bioflocculant BF-PX353943 demonstrated the lowest removal efficiency of 71.38% on turbidity reduction. However, bioflocculant BF-PP809651.1 illustrated a statistically similar removal efficiency (*p* value > 0.05) with polyaluminium chloride (PAC) and aluminium sulphate on turbidity reduction. The chemical oxygen demand (COD) removal efficiencies of bioflocculants BF-PX353943 (73.33%) and BF-PP809651.1 (76.00%) were comparably similar to that of aluminium sulphate (*p* > 0.05), but significantly lower than that of PAC (82%).

## 3. Discussion

In a natural environment, bacteria release bioflocculants as a defence mechanism due to abiotic and biotic stress [23,24]. Recreational ponds are the least untapped niches for bioflocculant producers. Therefore, exploration of these environments is crucial because it might lead to the discovery of novel bioflocculant bacteria with high bioflocculant production yields and flocculating activities. In this study, the low number of bioflocculant producers meant that the physicochemical conditions of the fishpond water did not support their growth and prevalence. The findings were contrary to those obtained by Luo et al. [25], where 46 isolates were promising bioflocculant producers.

The two potential bioflocculant producers were identified as *K. michiganensis* PX353943 and *K. pasteurii* PP809651.1. The genus *Klebsiella* consists of Gram-negative rod-shaped bacteria belonging to the family *Enterobacteriaceae* [26]. Generally, *Klebsiella* species are known to encompass a wide range of ideal strains for bioflocculant production. For instance, a study by Ma et al. [27] demonstrated that *Klebsiella* sp. OS-1 was able to synthesise sufficient bioflocculants. Notably, *Klebsiella pneumoniae* stands out among *Klebsiella* species as the most-reported potent bioflocculant producer. For instance, Luo et al. [28], Nakata and Kurane [29], Zhong et al. [30], Nie et al. [31], and Zhao and Zhou [32] have reported bioflocculant-producing *K. pneumoniae* strains. Nevertheless, to date, no study has reported specifically on *K. michiganensis* and *K. pasteurii* as bioflocculant producers. This suggests the potential to have a distinct diversity of bioflocculant-producing strains that might have potential for industrial applications.

In this study, PKS-1 and NRPS were not detected in either *K. pasteurii* PP809651.1 or *K. michiganensis* PX353943, implying that they were not involved in the bioflocculant production of these isolates. Only the *KSα* gene cluster, which encodes PKS-II, was successfully detected in the bioflocculant-producing bacteria *K. michiganensis* PX353943 and *K. pasteurii* PP809651.1. PKS-II is predominantly reported to be associated with the biosynthesis of secondary metabolites, particularly antibiotic, antifungal, and antitumor agents [33]. Even though there is no clear correlation between bioflocculant synthesis and PKS-II, there are several studies that have reported on bioflocculants with antimicrobial activity. For example, the polysaccharide-based bioflocculant (PBB) produced by *Bacillus subtilis* F9 exhibited antibacterial properties against all of the pathogenic bacterial strains with which it was tested [34]. Additionally, in a study conducted by Muthulakshmi et al. [35], the bioflocculants produced by two bioflocculants, namely, EB-EPS and B1-EPS, produced by *Enterobacter* sp. and *Bacillus* sp., also displayed antibacterial activity. This research-based evidence suggested that bioflocculant producers such as *K. michiganensis* PX353943 and *K. pasteurii* PP809651.1 can harbour and express a gene cluster encoding PKS-II. The presence of PKS-II highlights the metabolic versatility of these bacterial strains, which is ideal for a wide range of industrial applications.

The eps gene cluster encodes the biosynthesis of extracellular polymeric substances (EPS), which are the key components of bioflocculants. In this study, a gene from this cluster, epsJ, was not detected in either *K. pasteurii* PP809651.1 or *K. michiganensis* PX353943. Analogously, the epsJ gene was detected in *Streptococcus thermophilus* IP6756 [36]. However, the epsH gene was successfully detected in *K. pasteurii* PP809651.1 only. The epsH is known to encode glycosyltransferases, enzymes that catalyse the transfer of sugar moieties to growing polysaccharide chains, which is a key step in EPS biosynthesis. According to Chen et al. [13], epsH plays an essential role in the formation of the polysaccharide repeating units that make up the bioflocculant structure, utilising diverse sugar substrates depending on the bacterial species and environmental conditions. The detection of the glycosyltransferase-encoding gene in *K. pasteurii* PP809651.1 strongly confirmed the hypothesis that they produce polysaccharide-based bioflocculants and that the biosynthetic pathway is at least partially conserved across different genera. The presence of epsH also highlighted the potential for targeted genetic and metabolic engineering strategies. By manipulating this key EPS-encoding gene, it may be possible to enhance not only the yield of bioflocculants but also to tailor their structural complexity and functional performance. For example, a study conducted by Liu et al. [10] showed that overexpression of epsB, a gene within the eps gene cluster, can significantly enhance both the yield and performance of bioflocculants.

The inoculum size of 3% was a potent stimulant for bioflocculant production by *K. michiganensis* PX353943; this can be attributed to the balance between bacterial growth, metabolic activity, and nutrient availability. The 3% inoculum volume maintained sufficient active growth of cells while ensuring an adequate nutrient supply for bioflocculant synthesis. An inoculum size of less than 3% led to poor bioflocculant production, possibly because of the prolonged lag phase [37]. Furthermore, the observed drop in bioflocculant production at an inoculum size greater than 3% was due to niche overlapping, which inhibited the growth of the bacterium [38]. These findings are analogous to those reported by Wang et al. [39], where *Klebsiella mobilis* had a preferred inoculum volume of 5% for maximum bioflocculant production.

The peak bioflocculant production by *K. pasteurii* PP809651.1 was achieved with ab inoculum size of 1%. A lower cell density (1%) was ideal for gradual assimilation of nutrients, supporting steady multiplication of microbial cells and prolonged bioflocculant production. This aligned with the experimental findings by Srinivasan et al. [40], where bioflocculant production by *Klebsiella pneumoniae* was at its peak at the inoculum size of 1%. Large inoculum sizes often lead to the excessive proliferation of microbial cells and, consequently, the depletion of essential nutrients before bioflocculant production [41]. Hence in this study, there was a decrease in bioflocculant production with an increase in inoculum size (>1%). Generally, inoculum sizes ranging within 1–5% are of economic preference [42]. Thus, *K. michiganensis* PX353943 and *K. pasteurii* PP809651 can be regarded as being economic in terms of production costs.

The nutritional sources such as carbon and nitrogen sources are not only limited to influencing the yield of the bioflocculants but also the structure and composition of bioflocculants [43]. In the study, galactose was used in all subsequent experiments as it was the most preferred potent stimulant for both *K. michiganensis* PX353943 and *K. pasteurii* PP809651.1. Both strains were able to assimilate galactose for energy, growth, and bioflocculant production efficiently. Hence, galactose served as a precursor for the biosynthesis of the bioflocculants. The metabolism of galactose is linked with one of the major sugar pathways, the Leloir pathway, which facilitates catabolism of galactose via conversion to uridine diphosphate (UDP)-galactose [44]. The sugar phosphate UDP-galactose is one of the crucial components for the biosynthesis of exopolysaccharides [45], hence activation of the Leloir pathway may increase bioflocculant production.

Amino acids are characterised by the presence of an amino group. Several studies have adopted the use of amino acids as a nitrogen source for microbial bioflocculant production due to the presence of nitrogen at the amino group. In bioflocculant production, the amino acids offer a double role; they serve as a bioflocculant precursor and source of nitrogen. In this study, *K. michiganensis* PX353943 effectively assimilated tryptophan and produced its peak amount of bioflocculants. This implies that tryptophan acted as a key building block for a protein component of the bioflocculants. Furthermore, a mixture of nitrogen sources, including yeast extract, urea, and ammonium sulphate, also yielded slightly similar (*p* > 0.05) bioflocculant production as tryptophan. This suggests that *K. michiganensis* PX353943 has specialised metabolic pathways to assimilate a wide range of nitrogen sources to synthesise bioflocculants. These findings agree with the observations by Yue et al. [46], where *Klebsiella* sp. MYC effectively assimilated similar complex nitrogen sources (yeast extract, urea, and ammonium sulphate) for the biosynthesis of bioflocculants.

*K. pasteurii* PP809651.1 yielded its peak bioflocculant production when a complex nitrogen source (yeast extract, urea, and ammonium sulphate) was used as the nitrogen source. This suggests that *K. pasteurii* PP809651.1 has complementary metabolic pathways for all the components in this complex nitrogen source. In the complex nitrogen source, urea served as a readily available and assimilable source for the rapid growth of the bacteria [47]. The yeast extract was essential for providing the bacterium with growth factors, such as trace elements, sugars, vitamins, peptides, and amino acids, which stimulated its cellular metabolism and enzymatic activities crucial for the biosynthesis of bioflocculants [48]. Furthermore, ammonium sulphate might have contributed to the steady release of nitrogen, which helped to maintain normal cellular function during the fermentation. Hence, the combination of these nitrogen sources likely created a nitrogen-balanced environment that supported robust metabolic activity for biosynthesis of the bioflocculant.

The initial pH of the growth medium determines the net charge of microbial cells and oxidation–reduction potential [49]. The strain *K. michiganensis* PX353943 produced its maximum amount of bioflocculants when the initial pH of the medium was 5, implying that, at pH 5, the enzymes were functioning efficiently. Acidic conditions are known to stimulate stress responses in microorganisms, including the overexpression of genes responsible for the production of extracellular polymeric substances (EPS) (bioflocculant) [50]. Hence, at pH 5, *K. michiganensis* PX353943 was liable to secrete the bioflocculants as a defensive mechanism to protect itself from the pH stress. Furthermore, the initial pH of 5 might have influenced the effective uptake and availability of nutrients and ions crucial for microbial growth and metabolism [51].

The neutral condition (pH 7) was the most favourable for biosynthesis of bioflocculant production in *K. pasteurii* PP809651.1. This suggests that the neutral pH provided a conducive biochemical environment for the enzymes involved in bioflocculants to function effectively. Bioflocculant production decreased significantly when the medium pH was altered from the neutral pH. This can be attributed to the denaturation of the enzymes responsible for bioflocculant production or the reduced affinity of the substrates towards the enzymes. Therefore, the neutral pH promoted rapid cell division and robust metabolic activity, which are the core prerequisites for bioflocculant production [52]. These results are comparable with those reported by Yin et al. [53], whereby *Klebsiella* sp. ZZ-3 depicted identical peak bioflocculant production at pH 5 and pH 7.

Optimum temperatures are ideal for maximum growth and bioflocculant production. In this study, both strains, *K. michiganensis* PX353943 and *K. pasteurii* PP809651.1, preferred 35 °C as the optimum temperature. In the literature, *Klebsiella* species are generally documented as mesophilic bacteria, with optimal growth temperatures between 35 and 37 °C [54]. Therefore, the results of this study supported this notion. The findings were not surprising as Zhang et al. [55] and Salehizadeh and Shojaosadati [56] had previously highlighted that the enzymes responsible for bioflocculant production are generally active within a mesophilic range (25 to 37 °C). Thus, the metabolic activity and enzymatic activity of *K. michiganensis* PX353943 and *K. pasteurii* PP809651.1 were operating adequately to synthesise the bioflocculants. At the optimum temperature (35 °C), the bacterial cells were capable of adequate uptake of nutrients, and cellular respiration was heightened to synthesise abundant ATP (energy). The excess ATP was channelled into biosynthetic pathways; hence, there was increased biosynthesis of the bioflocculants. Temperatures below or above the optimum temperatures (35 °C) might have denatured the biosynthetic enzymes or halted the metabolic activities of bacteria, consequently lowering bioflocculant production [57]. In addition, thermal stress is capable of shifting gene expression away from the biosynthesis of bioflocculants and possibly rupturing the cell membrane of bacterial cells and damaging biosynthesis pathways. These findings correlate with those of Fang et al. [58], whereby *Klebsiella* sp. 59L displayed an identical peak bioflocculant production at 35 °C.

Bioflocculant production is strain-dependent, and it occurs during various growth phases among different strains. In this study, *K. michiganensis* PX353943 yielded maximum bioflocculant production at the beginning of the stationary phase (after 60 h). The cells multiplied rapidly from 0 to 48 h, peaking at 48 h, which indicated the end of the exponential phase. Bioflocculant production also increased with cell density but peaked at 60 h, suggesting that the biosynthesis of the bioflocculants was growth-associated. According to Agostini-Costa et al. [59], secondary metabolites are produced abundantly during the transition from the exponential growth phase to the stationary phase, further affirming the findings in this study. The stationary phase is characterised by a lack of nutritional sources; under such stress, the microorganisms tend to redirect their metabolism towards the biosynthesis of EPS to offer themselves protection against environmental stress. These results are in agreement with the findings of Luo et al. [28], whereby *Klebsiella pneumoniae* YZ-6 had a maximum bioflocculant production after 60 h of incubation, which was a transition point from the exponential phase to the stationary phase. In this study, the output of bioflocculants was carried out using closed batch fermentation, meaning that after the stationary phase, the death phase proceeded. During the death phase, nutrients are completely exhausted, cells undergo autolysis, and toxic metabolic by-products accumulate, hence the decrease in bioflocculant production and cell density post 60 h. For industrial applications, strains with high bioflocculant production within a short time (≤72 h) are mostly preferred since they result in low production costs [42]. Thus, the bioflocculant production of *K. michiganensis* PX353943 should be considered to be economically friendly.

The strain *K. pasteurii* PP809651.1 also demonstrated synchronisation between the kinetic growth of the cells and the biosynthesis of the bioflocculant. The peak bioflocculant production of this strain was observed at the late stationary phase, after 96 h. In a closed batch culture, this phase is usually associated with nutrient depletion, which translates into enhanced metabolic stress, which then triggers the production of secondary metabolites such as bioflocculants. These findings contradict those observed by Nguyen et al. [60], whereby *Klebsiella variicola* BF1 yielded peak bioflocculant production after 20 h of incubation.

*K. michiganensis* PX353943 and *K. pasteurii* MS18 produced promising bioflocculant yields. According to our literature search, the strain *K. pasteurii* PP809651.1 holds the record for the highest bioflocculant yield of 7.9 g/L among *Klebsiella* species. However, the existing literature documents that *Klebsiella* species have emerged as prominent high-yield bioflocculant producers. For example, *K. variicola* BF1 achieved the highest yield of 7.5 g/L [60], whereas *K. pneumoniae* H12 produced 3.0 g/L of extracellular polysaccharide bioflocculant [29]. *Klebsiella* M1 also yielded 3.91 g/L of bioflocculant under optimised extraction conditions [61]. To sum up, based on this literature search, it is evident that *K. pasteurii* PP809651.1 is a good bioflocculant producer and has potential applicability industrially.

The bioflocculants BF-PX353943 and BF-PP809651 demonstrated similar COD RE with aluminium sulphate and the bioflocculant BF-PP809651.1 further illustrated similar turbidity reduction with both PAC and aluminium sulphate, implying the bioflocculants have potential to serve as effective alternatives to aluminium sulphate and PAC in wastewater treatment. The findings agree with other reported findings whereby bioflocculants achieved significantly similar %RE on diverse wastewater parameters [62,63].

## 4. Materials and Methods

### 4.1. Sample Collection

The grab water samples were collected from a fishpond located in the University of Limpopo, Limpopo province, Republic of South Africa (23°52′56.4″ S 29°44′17.4″ E). The water samples were stored temporarily on ice and transported to the Microbiology laboratory at the Department of Biochemistry, Microbiology and Biotechnology at the University of Limpopo for further experiments.

### 4.2. Enrichment of Bioflocculant-Producing Bacteria

Nutrient broth was used as an enrichment medium. Briefly, the nutrient broth was prepared following the manufacturer’s instructions. Subsequently, the homogenised (by shaking) water samples from three (3) sampling sites were pipetted aseptically into three different flasks of nutrient broth. The flasks were then incubated in a shaking incubator (Orbital shaker supplied by Lasec (Pty) Ltd., Midrand, South Africa) at 30 °C using the shaking speed of 150 revolutions per minute (rpm) for 24 h.

### 4.3. Serial Dilution and Isolation of Bioflocculant-Producing Bacteria

The 10-fold serial dilution of the culture broth of the enrichment medium was performed using a sterile 0.85% saline solution. In short, 1 mL of the aliquot was pipetted aseptically into 9 mL of saline solution. In terms of the isolation of bioflocculant-producing bacteria, the production agar medium plates were prepared by dissolving the following components in 1 L of filtered pond water; glucose (20 g), K_2_HPO_4_ (5 g), K_2_PO_4_ (2 g), urea (0.5 g), yeast extract (0.5 g), MgSO_4_·7H_2_O (0.2 g), (NH_4_)_2_SO_4_ (0.2 g), NaCl (0.1 g) bacteriological agar (20 g), methyl red dye (100 mg), and cycloheximide (0.04 g). The medium was sterilised by autoclaving at 121 °C for 15 min. Thereafter, the spread plate technique was employed, whereby 100 µL of serially diluted and undiluted samples were pipetted and spread evenly on the production agar medium using a hockey stick. The plates were then incubated at 30 °C for 7 days. The colonies were selected randomly per their colour, size, and morphology. The selected colonies were purified by sub-culturing them twice on the same agar medium.

### 4.4. Screening of Bioflocculant-Producing Bacteria

#### 4.4.1. Fermentation

The screening of bioflocculant production was carried out according to the method described by Kurniawan et al. [64]. The purified bacteria were cultured in a broth production medium composed of the same ingredients as the ones used for the isolation process except the bacteriological agar. The medium was sterilised by autoclaving at 121 °C for 15 min. The bacterial isolates obtained were adjusted to the McFarland standard (1 × 10^8^ colony forming units/mL). Thereafter, the pure colonies were suspended in a sterile 0.85% saline solution and homogenised by vortexing. Subsequently, the bacterial suspension was adjusted to a McFarland standard by reading its absorbance (0.085 to 0.1 nm) using a spectrophotometer (Genesys 10 UV spectrophotometer, Thermo Fisher Scientific (Pty) Ltd., Waltham, MA, USA) at the wavelength of 625 nm. Then, 1% of the culture adjusted to a McFarland standard was pipetted aseptically in a broth production medium and grown at 30 °C in a shaking incubator at 150 rpm for 5 days. Thereafter, 2 mL of the culture broths were centrifuged at 10,000 rpm for 10 min at 4 °C, and the resulting supernatant was used to determine the flocculating activity (FA).

#### 4.4.2. Determination of FA

FA was determined following the procedure described by Maliehe et al. [65]. Two millilitres of the supernatant of each isolate were used to determine the FA. Briefly, kaolin clay solution (3 g/L) was prepared using deionised water and its pH was adjusted to pH 7 using 0.1 M HCl and 0.1 M NaOH. Thereafter, 3 mL of 1% of CaCl_2_ and 2 mL of the supernatant were added into 95 mL of kaolin clay solution in a 250 mL flask. The mixture was agitated vigorously for 1 min at 200 rpm and then gently for two minutes at 50 rpm. Then, the mixture was poured into a 100 mL calibrated measuring cylinder and then left to settle for 5 min at room temperature. A control experiment was also carried out similarly in a separate flask using 2 mL of sterile distilled water instead of the supernatant. Subsequently, the clarifying top layer from tests measuring cylinders was drawn using a micropipette, and its OD was measured at 550 nm using a spectrophotometer (Genesys 10 UV spectrophotometer, Thermo Fisher Scientific (Pty) Ltd., Waltham, MA, USA). The distilled water was used as the blank. The percentage flocculating activity (%FA) was calculated using Equation (1):

%FA = [(A_0_ − A_1_)/A_0_] × 100, (1)

where A_0_ and A_1_ are the OD readings of the control and test sample, respectively.

### 4.5. Molecular Identification of the Bioflocculant-Producing Bacteria

The identification of the isolates was outsourced to Inqaba Biotechnical Industries (Pty) Ltd., Pretoria, South Africa. The isolates were identified using a molecular technique based on 16S rDNA and *rpo*B gene amplification by polymerase chain reaction (PCR). Briefly, the genomic DNA of the bacteria was extracted using the Quick-DNA™ Fungal/Bacterial Miniprep Kit (Zymo Research Corp, Catalogue No. D6005, Irvine, CA, USA) following the manufacturer’s instructions. The purity and concentration of the extracted DNA were determined using the NanoDrop ONE spectrophotometer (Thermo Fisher Scientific, Waltham, MA, USA). The 16S rDNA gene of the bioflocculant-producing bacterium, isolate 18, was amplified using the 16S-27F primer (5′-AGAGTTTGATCMTGGCTCAG-3′) and 16S-1492R primer (5′-CGGTTACCTTGTTACGACTT-3′). Similarly, the *rpo*B gene of isolate MS7 was amplified using the universal primers Kleb-RpoB (5′-GGCGAAATGGCWGAGAACCA-3′) and Kleb-RpoB R (5′-GAGTCTTCGAAGTTGTAACC-3′). The general PCR conditions were set as follows: the initial denaturation was carried out at 94 °C for 5 min, followed by 35 repetitive cycles where denaturation was carried out at 94 °C for 30 s, annealing at 50 °C for 30 s, and extension at 68 °C for 1 min. The final extension was carried out at 68 °C for 10 min. Thereafter, the amplicons were held at 4 °C upon completion. The integrity of the PCR amplicons was visualised on a 1% agarose gel stained with EZ-vision^®^ Bluelight DNA Dye (AMRESCO LLC, Solon, OH, USA). The amplicon fragments were enzymatically purified using the ExoSAP procedure (NEB M0293L; NEB M0371) (New England Biolabs (NEB), Ipswich, MA, USA). The purified amplicons were sequenced using ZR-96 DNA Sequencing Clean-up Kit™, Catalogue No. D4050 (Zymo Research Corp., Irvine, CA, USA) and sequenced in the forward and reverse directions using, BrilliantDye™ Terminator Cycle Sequencing Kit V3.1, BRD3-100/1000 (NimaGen B.V., Nijimegen, Gelderland, The Netherlands), and then analysed using the ABI 3730xl Genetic analyser (Applied Biosystems, Thermo Fisher Scientific, Fostcity, CA, USA). The CLC Bio Main Workbench (QIAGEN, Aarhus, Denmark) was used to assemble the forward and reverse sequencing reads to form a consensus sequence for each sample. The Basic Local Alignment Search Tool (BLAST) (https://blast.ncbi.nlm.nih.gov/Blast.cgi, accessed on 21 October 2025) programme, accessible through the National Centre for Biotechnology Information (NCBI) database, was used to find the closest bacterial species through the homology of the sequences [66,67].

### 4.6. Evaluation of BGC and Enzymes Responsible for Bioflocculant Production

The previously extracted DNA samples (10–50 ng/mL) were used to identify specific BGCs such as PKS-1, PKS-II, and NRPS and enzyme glycosyltransferase, which might be involved during bioflocculant production by the selected bacterial isolates. About 25 μL of the PCR mixture was prepared using the PCR mixture and consisted of 12.5 μL of NEB One Taq 2X Master Mix (New England Biolabs (NEB), Ipswich, MA, USA) with a standard buffer, 2 × 1 μL of forward and reverse primers, 2 μL of the DNA samples, and 8.5 μL of nuclease-free water. The specific primers and PCR conditions outlined in Table S1 were used for amplification of the extracted DNA. The PCR products were then subjected to gel electrophoresis and the gel images were captured using GeneSnap software (Version 7.02) [68].

### 4.7. Optimisation of Medium Composition and Culture Conditions

The optimisation was performed by manipulating the original composition of the production medium (carbon and nitrogen sources) and growth conditions (inoculum volume, initial pH of the production medium, temperature, and cultivation time) using the a one-factor-at-a-time method. Before the investigation of each assay, the seed culture of the bacterial strains was prepared using a nutrient broth and adjusted to the McFarland standard (1 × 10^8^ colony forming units/mL).

#### 4.7.1. Effect of Inoculum Size on Bioflocculant Production

The dissimilar inoculum sizes were used to evaluate their effect on bioflocculant production in accordance with the method described by Tsilo et al. [49]. Briefly, dissimilar inoculum sizes (1–5% (*v*/*v*)) of the bacterial cultures at the exponential growth phase were aseptically inoculated into the sterilised broth production medium. Therefore, they were incubated at 30 °C in a shaking incubator (Orbital shaker supplied supplied by Lasec (Pty) Ltd., Midrand, South Africa) at 150 rpm for 5 days. Afterwards, 2 mL of the culture broth was centrifuged at 10,000 rpm, 4 °C for 10 min and used to assess the flocculating activity as outlined previously.

#### 4.7.2. Effect of Carbon Sources on Bioflocculant Production

The influence of carbon sources on bioflocculant production by the selected bacteria was evaluated using the method outlined by Nkosi et al. [69]. The broth production medium was prepared by substituting the glucose (20 g/L) from the original production medium with different carbon sources (sucrose, starch, xylose, lactose, and galactose) of the same amount, while other medium components were kept constant. The optimum inoculum size of the bacterial cultures at the exponential growth phase were aseptically pipetted into the production medium and incubated at 30 °C in a shaking incubator (Orbital shaker supplied supplied by Lasec (Pty) Ltd., Midrand, South Africa) at 150 rpm for 5 days. Thereafter, the culture broth (2 mL) was centrifuged at 10,000 rpm, 4 °C for 10 min and was used to determine the flocculating activity, as outlined before.

#### 4.7.3. Effect of Nitrogen Sources on Bioflocculant Production

The effect of nitrogen substrates on bioflocculant production was evaluated using the method elucidated by Ugbenyen et al. [70]. The mixture of nitrogen sources (urea, yeast extract, and (NH_4_)_2_SO_4_) previously used in the original production medium was substituted with 1.2 g/L of different nitrogen sources (urea, yeast extract, nitrate, tryptophan, (NH_4_)_2_SO_4_, NH_4_Cl, malt extract, casein, and NH_4_NO_3_) and other components were kept unchanged. The optimum inoculum size of the bacterial cultures at an exponential growth rate were then pipetted aseptically into the production medium and fermented at 30 °C in a shaking incubator (orbital shaker incubator from Lasec (Pty) Ltd., Midrand, South Africa) at 150 rpm for 5 days. After incubation, 2 mL of the culture broth was centrifuged (10,000 rpm, 4 °C for 10 min) and used to assess the flocculating activity, as previously outlined earlier.

#### 4.7.4. Effect Initial of pH on Bioflocculant Production

The effect of the initial pH of the production medium was determined following the method used by Abu Tawila et al. [71]. Briefly, the initial pH of the modified production medium (with the best pre-determined carbon and nitrogen source) was adjusted in a range of 3 to 12 using 0.1 M HCl and 0.1 M NaOH. Thereafter, the optimum inoculum sizes of the bacteria at the exponential growth phase were pipetted aseptically into different flasks of the production medium of different pH and incubated at 30 °C in a shaking incubator (Orbital shaker supplied by Lasec (Pty) Ltd., Midrand, South Africa) at 150 rpm for 5 days. Then, 2 mL of the culture broth was centrifuged (10,000 rpm, 4 °C for 10 min) and utilised to determine the flocculating activity as described previously.

#### 4.7.5. Effect of Temperature on Bioflocculant Production

The effect of the culture temperature on the biosynthesis of bioflocculant was investigated according to Zhao et al. [32]. A production medium with the best pre-determined carbon and nitrogen source and adjusted to the ideal pH was inoculated with the optimum inoculum sizes of the bacteria. It was then incubated at different temperatures (20–40 °C) in shaking incubators (Orbital shaker supplied by Lasec (Pty) Ltd., Midrand, South Africa) at 150 rpm for a period of 5 days. Afterwards, the culture broths were centrifuged at 10,000 rpm at 4 °C for 10 min and the flocculating activity was determined, as outlined previously.

#### 4.7.6. Effect of Incubation Time on Bioflocculant Production and Growth Rate

The effect of incubation time on bioflocculant synthesis and bacterial growth was evaluated according to the method described by Selepe et al. [9]. An optimised production medium was prepared and subsequently inoculated with the optimum inoculum sizes of the bacterial cultures at the optimum temperatures in a shaking incubator (Orbital shaker supplied by Lasec (Pty) Ltd., Midrand, South Africa) at 150 rpm. Thereafter, the bacterial culture broth (2 mL) was aseptically sampled at the interval of 12 h for a period of 108 h. Afterwards, the OD of the broth cultures were measured at 600 nm to determine the growth rate of the bacteria. Subsequently, the broth cultures sampled were centrifuged (10,000 rpm, 4 °C for 10 min) and used for evaluation of the flocculating activity, as outlined previously.

### 4.8. Extraction and Purification

The bioflocculants were extracted from the bioflocculant-rich broth in accordance with the technique used by Choi et al. [72]. The culture broths were separately centrifuged for 30 min at 8000 rpm. Thereafter, 2 volumes of absolute ethanol were added to the supernatant. The mixtures were thoroughly agitated and precipitated for 12 h at 4 °C. The precipitates were obtained by evaporating the solvent under laminar flow to obtain crude bioflocculants. The crude bioflocculants were then dissolved in autoclaved distilled water to produce solutions of 1% (*w*/*v*). One volume of a mixture of methanol and chloroform (1:2 *v*/*v*) was added and left at room temperature for 12 h. Afterwards, the supernatant was centrifuged (8000 rpm for 30 min, 4 °C) and air-dried under laminar flow to obtain purified bioflocculants.

### 4.9. Application of the Bioflocculants in Wastewater Treatment

The purified bioflocculants were used to treat wastewater from the Polokwane Wastewater Treatment Plant in Limpopo, South Africa. The pH of the wastewater was adjusted to pH 5 for bioflocculant BF-PX353943 and pH 7 for BF-PP809651.1 using 0.1 M HCl and 0.1 M NaOH. Chemical flocculants such as aluminium sulphate and PAC served as positive controls. The removal efficiencies of the flocculants on turbidity and COD were evaluated by the turbidimeter Hanna HI801-01 (Hanna Instruments, Woonsocket, RI, USA) and Spectroquant COD test kit (Merk KgaA, Darmstadt, Germany), following the manufacturers’ protocols. The RE was measured and expressed as percentage RE using the following formula:

%RE = (A_o_ − A)/A_o_ × 100, (2)

where A_o_ and A represented the values obtained before and after treatment, respectively [73].

### 4.10. Statistical Analysis

Experiments were conducted in triplicate. The means and standard deviations were determined using a one-way analysis of variance in Graph Pad Prism™ version 8.4.2. All *p*-values < 0.05 were considered significant, while *p*-values > 0.05 were considered insignificant. Also, Tukey’s test was utilised to verify significant differences (*p* < 0.05) among the means, where each mean was compared to every other mean.

## 5. Conclusions

In conclusion, screening and isolating bioflocculant producers from recreational fishpond water led to the discovery of bioflocculant-producing *K. michiganensis* PX353943 and *K. pasteurii* PP809651.1. Both bacteria possessed the PKS-II biosynthetic gene cluster, and *K. pasteurii* also revealed the presence of the epsH gene, which are responsible for bioflocculant production. At optimum conditions, *K. michiganensis* PX353943 demonstrated a maximum bioflocculant production with flocculating activity of 95% whilst *K. pasteurii* PP809651.1 showed maximum bioflocculant production with flocculating activity of 83%. Their bioflocculants demonstrated a high %RE (>70%) on COD and turbidity of the wastewater. For future studies, whole genome and transcriptomics analyses ought to be performed to understand the expressed genes, enzymes, and pathways involved during bioflocculant production. Moreover, detailed research into genetic engineering and production scaling are important. Lastly, optimisation of operational conditions and the mechanism of bioflocculation in wastewater ought to be evaluated to improve the removal efficiencies of the bioflocculants.

## Figures and Tables

**Table 1:** The effect of carbon and nitrogen sources on bioflocculant production by *K. michiganensis* PX353943 and *K. pasteurii* PP809651.1.

	Strain PX353943	Strain PP809651.1		Strain PX353943	Strain PP809651.1
Carbon Source	FA (%) ± SD	FA (%) ± SD	Nitrogen Source	FA (%) ± SD	FA (%) ± SD
Glucose	65 ± 0.036 ^a,b^	56 ± 0.037 ^a^	Urea	13 ± 0.003 ^a^	64 ± 0.003 ^a^
Galactose	74 ± 0.025 ^a^	79 ± 0.008 ^b^	Yeast extract	26 ± 0.130 ^a,b^	35 ± 0.025 ^c^
Sucrose	56 ± 0.022 ^b^	50 ± 0.075 ^a^	Tryptophan	80 ± 0.043 ^c^	52 ± 0.009 ^b^
Xylose	65 ± 0.146 ^a,b^	48 ± 0.041 ^a^	(NH_4_)_2_SO_4_	74 ± 0.046 ^c^	58 ± 0.029 ^a,b^
Lactose	65 ± 0.071 ^a,b^	44 ± 0.007 ^a^	Mixture (urea, yeast extract, and (NH_4_)_2_SO_4_)	76 ± 0.091 ^c^	79 ± 0.010 ^d^
Starch	–	67 ± 0.041 ^a,b^	NH_4_Cl	–	55 ± 0.058 ^a,b^
			Malt extract	–	51 ± 0.018 ^b^
			Casein	–	59 ± 0.029 ^a,b^
			NH_4_NO_3_	38 ± 0.018 ^b^	–

FA denotes flocculating activity; SD represents standard deviation and the letters (a, b, c, d) denote statistical significance at *p* < 0.05. Values sharing the same letter within a column are not significantly different (*p* > 0.05).

**Table 2:** Removal efficiency of the flocculants.

Flocculant	Turbidity Reduction (%)	COD Removal (%)
PAC	94.59 ^a^	82.00 ^a^
Aluminium sulphate	92.14 ^a^	76.67 ^b^
BF-PX353943	71.38 ^b^	73.33 ^b^
BF-PP809651.1	90.62 ^a^	76.00 ^b^

The superscripts denote statistical significance at *p* < 0.05. Values sharing the same letter are not significantly different (*p* > 0.05).

## Data Availability

The bacteria identified in this study are available from Genbank https://www.ncbi.nlm.nih.gov/nuccore/PP809651.1 (accessed on 21 October 2025) for *K. pasteurii* and https://www.ncbi.nlm.nih.gov/nuccore/PX353943 (accessed on 21 October 2025) for *K. michiganensis*. Their accession numbers are provided in this manuscript. The other datasets used during this study are available from the corresponding author on request.

## Supplementary Materials

The following supporting information can be downloaded at https://www.mdpi.com/article/10.3390/ijms262110559/s1.

## Abbreviations

The following abbreviations are used in this manuscript:

FA	Flocculating activity
BGC	Biosynthetic gene cluster
RE	Removal efficiency
UDP	Uridine diphosphate
OD	Optical density
NRPS	Non-ribosomal peptide synthetase
PKS	Polyketide synthase
COD	Chemical oxygen demand
PAC	Polyaluminium chloride
EPS	Exopolymeric substances
