# Supplementary material for: Regulatory Machinery of Bacterial Bioflocculant Synthesis and Optimisation and Assessment of Bioflocculation Efficiency in Wastewater
Source: Int J Mol Sci. 2025 Oct 30;26(21):10559. doi: 10.3390/ijms262110559 (PMC12609847; doi:10.3390/ijms262110559)
Supplement: Supplementary file 1 [file ijms-26-10559-s001.zip › ijms-3932505-supplementary.pdf]

Table S1. Primers and PCR conditions used to detect the BGCs.

Primer	Target	PCR conditions
KS F (5′–GTSCCSGTSSCRTGSSHYTCSA–3′)<br>KS R (5′-CGCTCCATGGAYCCSCARCA–3′)	PKS–1	94 °C initial denaturation for 4 minutes, followed by 32 cycles of denaturation at 94 °C for 1 minute, annealing at 56.9 °C for 30 seconds and extension at 72 °C for 1 minute and 30 seconds. Followed by a final extension at 72 °C for 10 minutes and held at 4 °C.
KS*α* F (5′–TSGCSTGCTTGGAYGCSATC–3′)<br>KS*α* R (5′–TGGAANCCGCCGAABCCGCT–3′)	PKS–II	94 °C initial denaturation for 4 minutes, followed by 32 cycles of denaturation at 94 °C for 1 minute, annealing at 61.1 °C for 30 seconds and extension at 72 °C for 1 minute and 30 seconds. Followed by a final extension at 72 °C for 10 minutes and held at 4 °C.
A3 F(5′–GCSTACSYSATSTACACSTCSGG–3′)<br>A7 R (5′–SASGTCVCCSGTSCGGTAS–3′)	NRPS	94 °C initial denaturation for 4 minutes, followed by 35 cycles. denaturation at 94 °C for 1 minute, annealing at 56.9 °C for 30 seconds, and extension at 72 °C for 1 minute and 30 seconds. Followed by a final extension at 72 °C for 10 minutes and held at 4 °C.
*eps*J F (5′–CCAATAGACAGGAAAAGCAACG–3′)<br>*eps*J R(5′–GGTTATGCCTTGGTTTATCTTGAC–3′)	Glycosyltransferase	The pre-heating was at 95 °C for 5 minutes, 40 cycles of 95 °C for 30 seconds, 95 °C for 5 seconds, 60 °C

*eps*H F (5′–AAACTACCACACCAGCACC–3′)<br>*eps*H R (5′–ATAAAAGTCAACCTATTTCAAAACAAGG–3′)	for 30 seconds, and a final extension at 72 °C for 5 minutes and held at 4 °C.
